# Counteracting the overestimation of effect size in mass-univariate brain-wide association studies using cross-validation

**DOI:** 10.1038/s41398-026-04529-x

**Published:** 2026-11-04

**Authors:** Janik Goltermann, Nils R. Winter, Marius Gruber, Lukas Fisch, Maike Richter, Dominik Grotegerd, Katharina Dohm, Susanne Meinert, Elisabeth J. Leehr, Joscha Böhnlein, Anna Kraus, Katharina Thiel, Alexandra Winter, Kira Flinkenflügel, Ramona Leenings, Carlotta Barkhau, Jan Ernsting, Klaus Berger, Heike Minnerup, Benjamin Straube, Nina Alexander, Hamidreza Jamalabadi, Frederike Stein, Katharina Brosch, Adrian Wroblewski, Florian Thomas-Odenthal, Paula Usemann, Lea Teutenberg, Julia-Katharina Pfarr, Andreas Jansen, Igor Nenadić, Tilo Kircher, Christian Gaser, Nils Opel, Tim Hahn, Udo Dannlowski

**Affiliations:** 1 Department of Psychiatry and Neurosciences, Campus Benjamin Franklin, Charité–Universitätsmedizin Berlin, Berlin, Germany; 2 German Center for Mental Health (DZPG), Site Berlin Potsdam, Germany; 3 Institute for Translational Psychiatry, University of Münster, Münster, Germany; 4 Institute for Machine Learning in Medicine (focus area psychiatry), University of Münster, Münster, Germany; 5 Department of Psychiatry, Psychosomatic Medicine, and Psychotherapy, Goethe University Frankfurt, University Hospital, Frankfurt, Germany; 6 Cooperative Brain Imaging Center - CoBIC, Goethe University Frankfurt, Frankfurt, Germany; 7 Institute for Translational Neuroscience, University of Münster, Münster, Germany; 8 Department of Clinical Psychology and Psychotherapy, Marburg University, Marburg, Germany; 9 Department of Neurology, Kepler University Hospital, Johannes Kepler University Linz, Linz, Austria; 10 Faculty of Mathematics and Computer Science, University of Münster, Münster, Germany; 11 Institute for Biomagnetism and Biosignalanalysis, Universitätsklinikum Münster, Münster, Germany; 12 Institute for Geoinformatics, University of Münster, Münster, Germany; 13 Institute of Epidemiology and Social Medicine, University of Münster, Münster, Germany; 14 Department of Psychiatry and Psychotherapy, Marburg University, Marburg, Germany; 15 Northwell Health, New Hyde Park, USA; 16 ORIGAMI Neuro Data Science Laboratory, McConnell Brain Imaging Centre, Montreal Neurological Institute, Faculty of Medicine and Health Sciences, McGill University, Montreal, QC Canada; 17 Core-Facility Brainimaging, Faculty of Medicine, Marburg University, Marburg, Germany; 18 Center for Mind, Brain and Behavior (CMBB), Universities of Marburg, Gießen and Darmstadt, Marburg, Germany; 19 Department of Psychiatry and Psychotherapy, Jena University Hospital, Jena, Germany; 20 Department of Neurology, Jena University Hospital, Jena, Germany; 21 German Center for Mental Health (DZPG), Site Jena Magdeburg Halle, Germany; 22 Department of Psychiatry, Medical School and University Medical Center OWL, Protestant Hospital of the Bethel Foundation, Bielefeld University, Bielefeld, Germany; 23 Center for Intervention and Research on Adaptive and Maladaptive Brain Circuits Underlying Mental Health (C-I-R-C), Site Jena Magdeburg Halle, Germany

**Keywords:** Neuroscience, Biomarkers, Scientific community

## Abstract

**Background:**

Brain-wide association effects are commonly overestimated in psychiatric neuroimaging research, leading to low replicability and generalizability of findings, and ultimately to poor translation to clinical utility. Cross-validation is currently not standardly used in univariate neuroimaging analyses but could pose a potential counteracting measure for this problem.

**Methods:**

We systematically evaluated the benefit of a cross-validation procedure to expose inflated effect size estimates in mass-univariate voxel-based analysis, using body mass index (BMI) as an example predictor for gray matter. A total of n = 3401 MRI scans from a psychiatric cohort were used to investigate the impact of applying cross-validation on effect size estimates across a range of sample sizes and across different localizations with a range of underlying pooled effect sizes.

**Results:**

BMI was negatively associated with gray matter density with a maximum underlying effect size of *R*²_p_ = 0.036 (in the full sample of n = 3401). However, without cross-validation, effects were substantially overestimated in small samples, with effect sizes ranging up to *R*²_p_ = 0.535. Even with an underlying null-effect, effect size estimates up to *R*²_p_ = 0.429 were reached in samples with n = 25. Applying cross-validation revealed that these inflated effects in small samples did not generalize to novel data. A minimum sample size of n = 100 was required for largest brain-wide effects to start generalizing, while samples with n = 400 were needed for smaller effects to generalize. This ‘point of initial generalizability’ was not reached for underlying null effects, even with the maximum available sample of n = 3401.

**Conclusions:**

Results suggest that cross-validation is an effective measure to expose overestimated effects in small samples and thus could help to counteract low replicability and generalizability in brain-wide mass-univariate association studies. Open-source code is provided for practical implementation.

## Introduction

Neuroimaging methods such as magnetic resonance imaging (MRI) have been used for several decades now to gain insights into the neurobiological underpinnings of psychological phenotypes. A plethora of scientific publications describes imaging-derived biomarkers of mental health disorders and perpetuates the hope of utilizing such methods to aid clinical decision-making [1]. However, recently the validity of findings involving relationships between neuroimaging and psychological phenotypes have been questioned due to accumulating reports of low replicability [2–6]. While such a replication crisis is not specific to neuroimaging research (e.g., for the field of psychology see Open Science Collaboration [7]), its contributing factors may be particularly potent in this research domain due to high analytic flexibility in combination with a large number of statistical tests, which is likely to amplify publication bias [8–10]. Relatedly, replicability is undermined by small samples [11] which lead to highly unstable effect size estimates [12–15]. Of note, such estimates are not systematically biased in terms of their direction and in principle would, on average, converge to the true effect if reported without selection. However, the greater sampling variability from small samples leads to more extreme values which leads to an overestimation of absolute effect size magnitude (independent of the direction of effects [4]). Filtering results for statistical significance further aggravates this problem, as only the most extreme estimates pass the threshold, resulting in systematic overestimation of reported effects. Paradoxically, this bias becomes stronger the more conservative the significance threshold, because increasingly extreme estimates are required to survive thresholding [4, 12].

As studies particularly in neuroimaging research frequently use small samples [16, 17], this likely further contributes to low replicability. Recently, Marek et al. demonstrated that even without publication bias, associations between psychological and brain phenotypes are not replicable unless thousands of participants are included in the analysis [4]. This seminal study has led to a vibrant discussion regarding replicability, sample size and effect size in the neuroimaging domain [1, 3, 18–21].

In summary, the outlined evidence emphasizes an urgent need for solutions to increase the replicability of psychiatric neuroimaging findings. One possible solution that has been suggested repeatedly is to validate brain effects in independent data via cross-validation [19, 22, 23]. While cross-validation methods are standardly used in multivariate brain analyses to identify and counteract overfitting and assure generalizability of models [24–27], they are rarely ever used on univariate brain effects, likely also because common neuroimaging analysis software packages do not offer options to conduct cross-validation. However, specifically in the domain of neuroimaging research, cross-validation methods may be useful even for univariate models as the outlined overestimation of effects can be seen as an overfitting of models in the face of high analytic flexibility in combination with a high number of tests in this domain. Thus, we investigate the utility of cross-validation for accurate estimation of effect size in univariate voxel-based brain analysis. Body mass index (BMI) is used as an example predictor due to previous reports of good replicability of BMI with brain structure, as well as its pivotal relevance for various mental disorders [28–31]. Due to reports of relatively large effect size estimates of the association between BMI and brain structure even in large samples (maximum effect size corresponding to approximately 2.7% explained variance [30]) this predictor is suitable to investigate the impact of cross-validation for a broader range of effect size, as compared to other predictors where effect size estimates across brain modalities have been shown to be considerably smaller [4, 32]. In order to evaluate the relevance of cross-validation for generalizability of effects sizes as a function of the sample size, we conducted our analyses across different sample sizes.

In light of the current lack of brain analysis software implementations regarding this matter, we provide open-source Python code to enable other researchers to apply cross-validation for voxel-based brain analysis.

## Method

### Participants

A total of n = 3401 participants were included from three independent German cohorts: the Marburg-Münster Affective Disorders Cohort Study (MACS; n = 1655), the Münster Neuroimaging Cohort (MNC; n = 722) and the BiDirect cohort (n = 1024). All three cohorts include individuals with major depressive disorder (MDD) and healthy controls (HC) free from any lifetime mental disorder diagnoses. General study methods, exclusion criteria and cohort characteristics were comprehensively described elsewhere (MACS: [33, 34]; MNC: [35, 36]; BiDirect: [37]). See supplements for an additional description of data exclusion steps and sample characteristics specific to the current analysis.

### Measures and procedure

BMI was calculated based on self-reported height and weight of participants. T1-weighted high-resolution anatomical brain images were acquired using 3 T MRI scanner in all three studies. For the MACS sample two different MRI scanners were used at the recruitment sites in Marburg (Tim Trio, Siemens, Erlangen, Germany; combined with a 12-channel head matrix Rx-coil) and Münster (Prisma, Siemens, Erlangen, Germany; combined with a 20-channel head matrix Rx-coil). Data from the MNC and BiDirect samples were acquired using the same Gyroscan Intera scanner (later with Achieva update; Philips Medical Systems, Best, The Netherlands). Image preprocessing was conducted using the CAT12-toolbox [38] (https://neuro-jena.github.io/cat/) using default parameters for all samples. Briefly, images were bias-corrected, tissue classified, and normalized to MNI-space using linear (12-parameter affine) and non-linear transformations, within a unified model including high-dimensional geodesic shooting normalization [39]. The modulated gray matter images were smoothed with a Gaussian kernel of 8 mm FWHM. Absolute threshold masking with a threshold value of 0.2 was used for all second level analyses as recommended for VBM analyses (https://neuro-jena.github.io/cat12-help/). Image quality was assessed by visual inspection as well as by using the check for homogeneity function implemented in the CAT12 toolbox.

### Statistical analysis

**Fig. 1:**
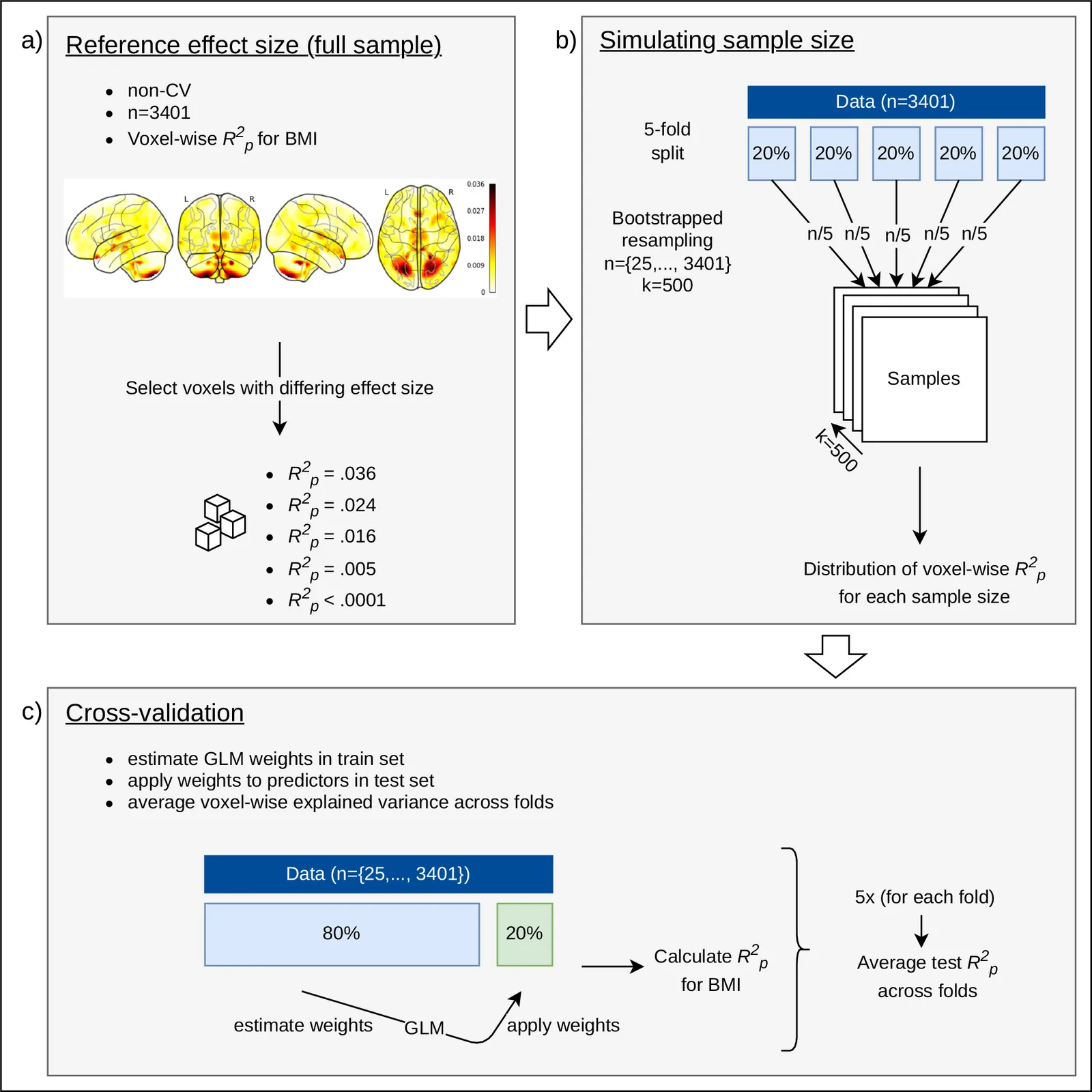
Analysis steps to obtain voxel-wise effect size estimates across sample sizes with and without cross-validation. *Note*. CV, cross-validation; BMI, Body Mass Index; GLM, general linear model.

We investigated the association between BMI and gray matter brain structure using an established mass-univariate voxel-based morphometry (VBM) approach. To that end, BMI was used as a predictor in a general linear model (GLM) to predict voxel-wise gray matter density. The following nuisance parameters were included in the model: age, sex, total intracranial volume (TIV) and four dummy-coded scanner variables to control for scanner hardware differences. Two-sided whole brain effects of BMI were tested and partial *R*^2^ (*R*^2^_p_) was used as a measure of effect size. To assess the impact of sample size and cross-validation on the estimation of voxel-based effect sizes the following steps were undertaken (for a schematic overview see Fig. 1):

1. Classical non-cross-validated analysis was conducted in the maximum available sample without bootstrapping (n = 3401). The resulting voxel-wise explained variance of BMI was used as an estimate for the realistic underlying effect size (in the following referred to as the *reference effect size*). Subsequently, voxels were selected covering a range of different representative effect sizes, including the voxel with the largest brain-wide reference effect size. In addition, the voxel with the smallest brain-wide reference effect size was selected to investigate effect size estimation based on an underlying null effect. An uncorrected significance threshold of p < 0.001 and extent threshold of k > 200 was used for visualization purposes of brain-wide associations.
2. Based on the findings of Marek et al. [4] we subsequently investigated the influence of sample size on the non-cross-validated effect sizes. Sample size was manipulated using samples of 18 different sizes: n = 25, 35, 50, 70, 100, 150, 200, 300, 400, 600, 800, 1000, 1300, 1600, 2000, 2400, 2900, 3401. For each preselected voxel a bootstrapped resampling distribution of effect size was obtained containing k = 500 bootstrap runs per sample size. For each sample size the mean effect size was calculated across all bootstrap runs, as well as a 95% confidence interval (CI).
3. Finally, the impact of cross-validation on effect size estimates in the selected voxels was investigated across samples created with the resampling procedure described above. To this end, 5-fold cross-validation was applied by estimating linear effects using the GLM described above within respective train sets and then applying the resulting linear coefficients to the respective test sets. To this end the resulting beta coefficients yielded by model estimation within the train set was applied to the design matrix (individual predictor values) of the test set (i.e., data new to the trained linear model). The predictive value of the BMI predictor was evaluated by calculating the explained variance (based on residuals) with and without BMI as a predictor in the model. Then the mean *R*^2^_p_ for BMI was calculated across the five test sets. This was used to quantify the extent to which the linear voxel-wise coefficients for BMI obtained from the train sets explain variance within unknown test data (i.e., generalization of effects to unseen data). Note that while *R*^2^_p_ usually has a range from 0–1, it can become negative (and even exceed -1) in this case due to the application of linear coefficients to unseen data which can result in an effect size lower than prediction by the mean within the same sample (normally the baseline reference corresponding to an effect size *R*^2^_p_ = 0). A *point of initial generalizability* was defined as the minimum sample size necessary to achieve positive effect sizes in the test sets (i.e., the lower bound of the 95% CI above *R*^2^_p_ = 0). This point of initial generalizability can be interpreted as the minimum sample size needed for linear effects of a given effect size to generalize to unknown samples in a 5-fold cross-validation framework. The 5-fold cross-validation was chosen over a higher number of folds to be able to simulate very small (but commonly used) samples sizes. In order investigate the impact of this cross-validation on effect size estimation across different sample sizes we applied the same bootstrap approach described above also to cross-validation-based effect size estimates, resulting in resampling distributions of average test set effect sizes for each sample size (95% CI were based on these bootstrap runs). To allow this combination of cross-validation and bootstrapping, we first conducted the 5-fold split of the total sample and then performed the bootstrapped resampling for each sample size by drawing with replacement from each split separately – thus only allowing replacement within one split but not across splits. This is necessary to avoid data leakage between train and test sets.

Notably, the reference estimate derived from the full sample is not independent of the effect size estimates obtained in the resampling analyses. Within the present framework it serves as a proxy for the underlying population effect, while the resampled subsets represent draws from this population to examine how effect size estimates behave across sample sizes. All analyses were conducted in Python (version 3.9.12) using the nilearn package (version 0.9.1) for voxel-based GLMs and k-Fold from the sklearn package (version 1.1.1) for cross-validation. The complete analysis code is provided online (https://osf.io/cy7fp/?view_only=a10fd0ee7b914f50820b5265f65f0cdb).

## Results

### Associations between BMI and gray matter – identification of reference effect sizes in n = 3401

**Fig. 2:**
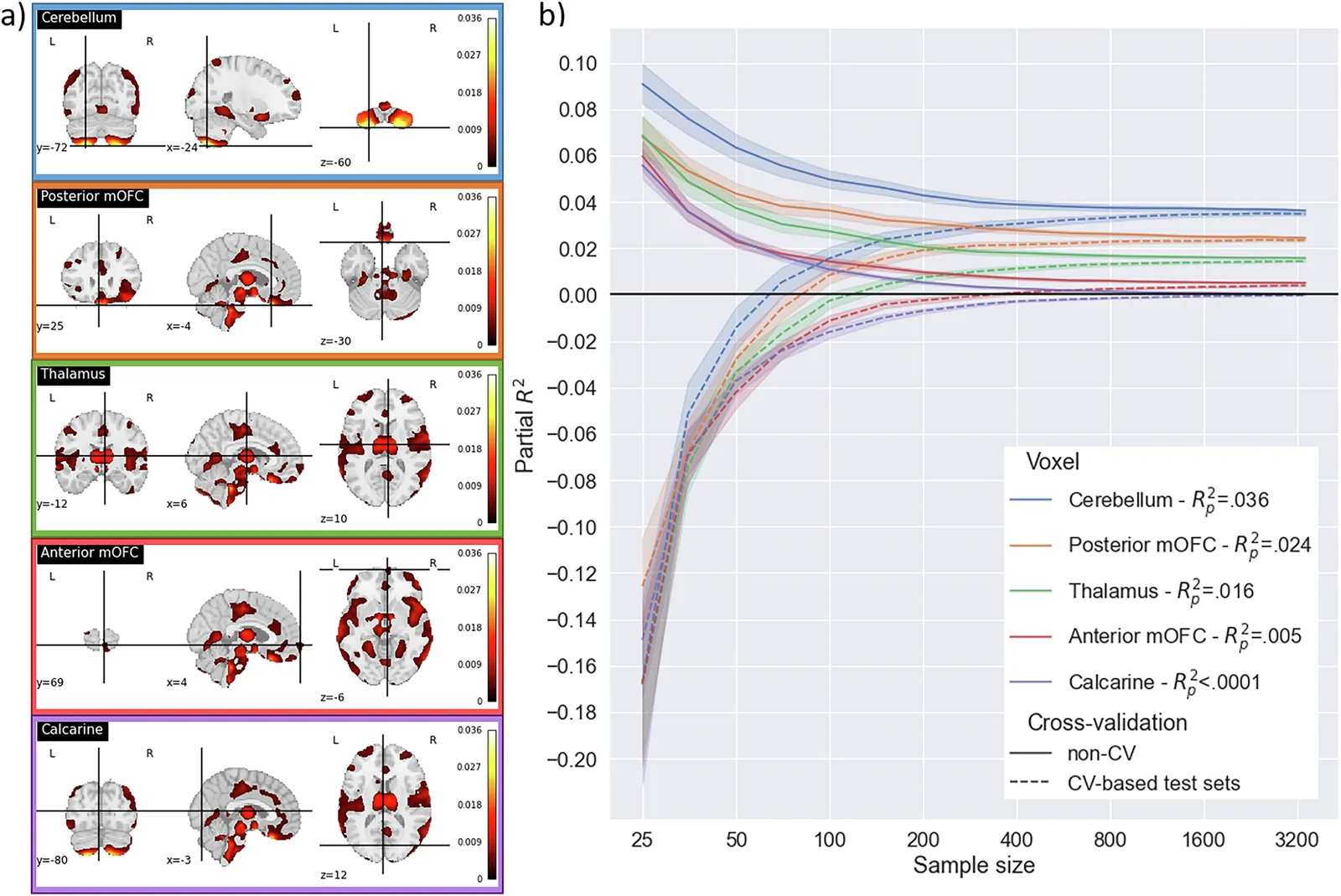
The association between Body Mass Index and gray matter density - selected voxel locations and their effect size estimates across sample sizes, with and without and cross-validation. *Note*. a) Shows non-cross-validated two-sided effect of BMI on gray matter density at a liberal uncorrected threshold of p < 0.001 in the maximum sample of n = 3401. The location of selected peak voxel coordinates in the cerebellum (largest brain-wide effect), posterior medial prefrontal cortex (mOFC), thalamus, anterior mOFC and calcarine (smallest brain-wide effect) are shown. Color bars represent R^2^_p_ values. b) Shows effect size estimates for the five different voxels across sample sizes (resampling with k = 500 per sample size). Non-cross-validated (non-CV) effect sizes are presented with solid lines and test set effect sizes with dotted lines. Please note that presented partial R² estimates express the magnitude of effects irrespective of their direction (with underlying effects constituting positive and negative correlations). For directional effect size estimates see supplementary Figure S2. Error bands represent the 95% confidence interval. Respective effect sizes of selected voxels in non-cross-validated analysis of full sample are presented in the legend (ranging from η²_p_ = 0.036 to η²_p_ < 0.0001).

The association between BMI and gray matter density in a non-cross-validated standard analysis is shown in Fig. 2a and supplementary Figure S1. Liberal thresholding at uncorrected p < 0.001, k > 200 resulted in widespread clusters across the brain. Effect size in significant voxels ranged up to *R*^2^_p_ = 0.036 with predominantly negative associations between BMI and gray matter (Figure S1). Peak voxel coordinates of significant clusters covering a wide range of effect sizes, as well as the voxel with the brain-wide smallest effect size were selected for further analysis (see Fig. 2a). The selected voxels from largest to smallest effects were located in 1) the cerebellum (*R*^2^_p_ = 0.036, p < 0.0001, x-24, y-72, z-60), 2) the posterior medial orbitofrontal cortex (mOFC; *R*^2^_p_ = 0.024, p < 0.0001, x-4, y25, z-30), 3) the thalamus (*R*^2^_p_ = 0.016, p < 0.0001, x6, y-12, z10), 4) the anterior mOFC (*R*^2^_p_ = 0.005, p < 0.0001, x4, y69, z-6) and 5) the calcarine (*R*^2^_p_ < 0.0001, p = 0.999, x-3, y-80, z12).

### The impact of sample size on non-cross-validated effect size estimation

Effect size was systematically overestimated in small samples in the non-cross-validated analysis. Averaged across all bootstrapped samples with size n = 25, effect size was inflated approximately 0.06 *R*^2^_p_ units for all five voxels, resulting in a 2.5-fold inflation for the voxel with the largest reference effect size (*R*^2^_p_ = 0.091 instead of *R*^2^_p_ = 0.036) and 11.9-fold inflation for the voxel with a reference effect size of *R*^2^_p_ = 0.005. Maximum effect size estimates went as high as *R*^2^_p_ = 0.734 in samples of n = 25. Even the voxel with the brain-wide lowest reference effect size (null-effect) reached a maximum effect size of up to *R*^2^_p_ = 0.429 (average *R*^2^_p_ = 0.056) in these samples with n = 25. Average estimates of effect size (as well as maximum estimates) decreased rapidly in a nonlinear fashion with increasing sample size.

Inspection of partial correlation coefficients (thus retaining the direction of effects) revealed substantial sign (type S error) and magnitude (type M error) instability of effects across bootstrapped samples, particularly in small sample sizes. For the voxel with the largest brain-wide reference effect size (negative association of r = -0.19 in full sample), associations ranging from a negative correlation r = -0.73 to a positive correlation r = 0.60 were found in samples with n = 25 (for presentation of directional effects see supplementary Figure S2).

Detailed summary statistics for effect size estimates across sample sizes and voxels are presented in supplementary Tables S2–6. While the average effect size estimate across bootstrapped samples may be informative, it should be noted that the distribution of estimates was highly skewed with some extreme outliers. This distribution across single runs from the resampling procedure is further visualized in supplementary Figure S3.

### The impact of cross-validation on effect size estimation

On average test set effect size estimates were descriptively lower compared to non-cross-validated effect sizes across all sample sizes and all voxels (this was true for 93.83% of bootstrapped samples across all sample sizes). This disparity was particularly strong in small samples where non-cross-validated effect sizes were largest while test set effect sizes were mostly negative (indicating no generalization of linear effects to unknown samples).

A *point of initial generalizability* was reached after n = 100 for the largest reference effect size, while larger samples were needed to reach this point for voxels with smaller effect size: n = 100 for *R*^2^_p_ = 0.024, n = 150 for *R*^2^_p_ = 0.016, and n = 400 for *R*^2^_p_ = 0.005. For the voxel with the minimum brain-wide effect size this *point of initial generalizability* was never reached, as expectable for an underlying null-effect. In fact, for this voxel even the mean and upper bound of the 95% CI were always below an effect size of zero at any sample size, when applying cross-validation, indicating effective protection against false-positives (see supplementary Tables S2–6).

Estimates derived from non-cross-validated analysis and from test sets using cross-validation approached convergence in larger samples. In samples with n = 3401, non- cross-validated and test set effect sizes differed only marginally (average difference across voxels: *R*^2^_p_ = 0.001). However, full convergence was never reached between non-cross-validated and test set effect size estimates, meaning that confidence intervals did not overlap, even at largest sample sizes. Detailed descriptive statistics for effect size estimates across sample sizes, voxels and cross-validation are given in supplementary Tables S2–6.

## Discussion

Using BMI as an example predictor variable, we investigated the utility of cross-validation for the accurate estimation of effect sizes of univariate brain-wide associations. Replicating previous findings [4], we find that effect size estimates are highly overestimated and unreliable in small samples if no cross-validation is applied. Furthermore, we demonstrate that cross-validation can be used to reveal poor generalizability to new data of these overestimated effects in small samples (indicated by negative effect size estimates). In larger samples, cross-validation-derived test set effect sizes start becoming positive, suggesting a *point of initial generalizability* that can be interpreted as the minimum sample size necessary to obtain model estimates that have any generalizability value in independent data. Importantly, sample sizes of several hundreds of participants may be sufficient to accurately estimate brain-wide associations, given sufficiently large underlying effect sizes. The cross-validation approach could facilitate a differentiation between artificially inflated large effects and robust ‘true’ large effects. The standardly implementation of cross-validation to assess the generalizability of brain-wide effects should be considered in addition to conventional reporting of significance and traditional effect size estimates. The utilization of cross-validation to facilitate generalizability of neuroimaging findings has been previously extensively investigated in the domain of multivariate machine learning analysis [40–42]. Furthermore, the application of cross-validation has been repeatedly demanded also in the context of univariate MRI analysis for example in the context of counteracting double-dipping and circular inference [22, 43, 44] but also more generally to assess the out-of-sample predictive utility of given effects and to counteract non-replicability [19, 23]. However, to the best of our knowledge, we are the first to systematically investigate the utility of cross-validation for effect size estimation in brain-wide univariate analysis and to provide a concrete tangible framework for others to apply to their analysis.

The presented overestimation of effect sizes in small samples falls in line with previous findings in the literature [12–15]. Marek et al. [4] demonstrated that even the largest brain-wide associations of compound cognitive and psychopathological variables with brain structure and function corresponded to less than 0.5% explained variance, which was inflated to up to approximately 40% explained variance in small samples. The authors conclude that thousands of individuals are needed for robust and replicable estimates in this research domain. While these findings are striking and important, the authors’ conclusion has been questioned by various scholars. The main critique is that true effect sizes may be considerably higher under ideal conditions leading to smaller sample sizes necessary for accurate estimation of effects [45]. In a study of over 1800 adults, Winter et al. [32] investigated univariate brain-wide, cross-modal brain differences between HC and lifetime MDD individuals and conclude that largest effect sizes go up to 1.7% explained variance. However, they further report that comparisons of HC with chronic or acute MDD individuals yield higher effects sizes of up to 2.7% explained variance (in n > 900). Further, studies suggest that effect sizes are larger in other diagnostic groups such as psychotic disorders [46]. Similarly, Repple et al. [27] present transdiagnostic structural connectome alterations, with largest effect sizes found in patients with schizophrenia, possibly rendering smaller samples sufficient to detect effects.

Our findings expand the results by Marek et al. [4] to a voxel-based analysis framework and to a broader range of effect sizes. Importantly, we provide evidence that for larger effect sizes – which can occur under specific conditions as outlined above – hundreds of participants could suffice to accurately estimate linear brain-wide effects. When smaller effect sizes are assumed, our findings are highly comparable to the findings by Marek et al. as we similarly find that an underlying reference effect size (based on the pooled sample) of approximately ~0.5% explained variance requires very large samples to obtain robust estimates of effects, although cross-validation may enable the robust detection of accurate estimates already in somewhat smaller samples (n > 400). The ongoing debate surrounding effect size and sample size in the neuroimaging domain stresses the importance of consistent reporting of effect sizes in publications, as well as interpreting effect sizes in the context of sample size.

While the above considerations could guide sample size planning of neuroimaging studies, a problem arises when the ‘true’ effect size is unknown (and effect size inflations in small samples make it particularly difficult to estimate this solely from existing literature). How can researchers know if an obtained effect size is accurate or inflated? Using a cross-validation framework, we demonstrate that the application of identified linear effects to unseen data can be utilized to identify inflated, non-generalizable effect sizes. Notably, this procedure can be applied in smaller studies to verify if a putative large effect size is robust and may warrant the use of a smaller sample. Importantly, our findings are in line with low test set performance merely reflecting an underpowered sample and not necessarily meaning that an effect does not exist [47]. In other words, even substantial effects of 3.6% explained variance can barely be differentiated from null effects in small samples based on cross-validation-derived effect sizes alone. However, the relative congruence between non-cross-validated and cross-validation-derived effect sizes can provide a strong argument for robust and replicable linear associations with realistic effect size estimates. Thus, calculating non-cross-validated and cross-validation-derived effect sizes combined with a thorough inspection of the similarity between the resulting estimates could be informative. Notably, while cross-validation decreases the probability of effect size inflation, it does not eliminate it. Even unrealistically high effect size estimates can in rare cases generalize to unseen test set data, particularly in small samples, as shown by some extreme outliers in our results.

While it is difficult to deduct a clear recommendation for sample sizes based on our cross-validation analyses, we propose that a *point of initial generalizability* can be defined as the sample size at which linear effects begin to explain variance in unseen data (in our framework defined as explanatory value > 0 in 97.5% of bootstrapped samples). Importantly, analogous to statistical significance, this criterion does not imply practical or clinical utility. In other words, it is arguably up for debate and strongly dependent on the research question whether a model should explain 1, 5 or 10% of variance in new data to be regarded as useful. However, we argue that a point of initial generalizability can be an informative starting point to address the important question of generalizability. This aspect could expand the traditional power analysis framework by the question of generalizability in addition to significance. In other words, while traditional power analysis answers the question of how large a sample needs to be for a given effect to become *significant*, our results open up the possibility for defining sample sizes necessary for linear effects to *generalize* to unseen data. Of note, similar concepts have been previously described, such as the point of stability (sample size where correlation estimates stabilize [15]). While conceptually related, the distinction is that the point of initial generalizability explicitly expresses the out-of-sample predictive utility.

Empirically, test set performances never reached the pooled-sample underlying effect size. This reflects a well-known pessimistic bias of k-fold cross-validation, because models are trained on only a subset of the data and therefore tend to show slightly lower out-of-sample performance than models fitted to the full dataset [48]. However, in large samples effect sizes became highly stable and barely differed whether cross-validation was applied or not. This finding implicates that if several thousands of individuals are available (e.g., due to consortia data), within sample k-fold cross-validation may barely alter the result, regardless of the underlying reference effect size of the pooled sample.

The current study entails several important limitations. Firstly, the concept of generalizability as used in the presented framework refers to predictive performance in unseen individuals within the same dataset rather than to generalizability across independently collected datasets. Thus, the *point of initial generalizability* should be interpreted as reflecting internal out-of-sample prediction within the studied population rather than external replication across different samples. Secondly, it is unclear whether our findings can be generalized to other cross-validation methods. While 5-fold cross-validation was chosen for the current analysis so that small samples of n = 25 could be included, other splits (e.g., 10-fold) could in principle also be suitable to be applied in voxel-based univariate brain analysis. While a systematic comparison of different cross-validation methods for the application to univariate voxel-based analysis is beyond the scope of this work, this may be a fruitful task for future studies. Further, it is open for discussion to what extent our findings are generalizable to other MRI modalities, such as functional MRI and parcellation-based brain analysis, as well as to predictors of other domains. We believe that generalizability to other voxel-based imaging modalities and other predictors is given, as the general pattern of results should not be specific to VBM effects of BMI. Regarding other predictors, there is no reason that they should show patterns qualitatively differing from BMI. Based on previous investigations and our results, the true underlying effect size is the main factor driving effect size overestimation and thus also the potential benefit of cross-validation. Regarding other imaging modalities, parcellation-based brain analysis approaches could also profit from cross-validation. However, cross-validation could possibly be particularly beneficial in settings that are at higher risk for overfitting statistical models. Such risk is likely to be higher in more complex models, and in settings with higher analytical flexibility and a higher number of statistical tests (the latter being higher in voxel-based as compared to parcellation-based analysis). Interestingly, cross-validation does not seem to ultimately protect from an inflation of effect size in all settings. It has been shown that in complex multivariate modelling of voxel-based associations with psychopathology, even cross-validation-based performance measures are inflated in underpowered studies [49]. In the same vein, we demonstrated that even cross-validation-derived effect sizes can be inflated in small samples although the probability of an overestimation of effects is lower as compared to non-cross-validated estimates (as outlined above).

In summary the utilization of cross-validation can contribute two major benefits: 1) identify if a study is underpowered and corresponding effect size estimates are likely to be highly inflated and 2) corroborate the accuracy of an effect size estimate of a sufficiently powered study (also for underlying null-effects). Thus, we propose that cross-validation procedures should be applied to foster replicability of neuroimaging research and facilitate accurate estimation of effect sizes. Further, we provide concrete steps for an application of cross-validation to mass-univariate voxel-based analysis, as well as corresponding open-source Python code.

## Supplementary information

Supplements

## Data Availability

Individual raw data is not published due to current EU data protection regulations and the sensitive nature of clinicalMRI data but can be made available in form of summary statistics or anonymized aggregation of voxel-wise data upon reasonable request to the corresponding author. All authors had full access to all study data.
